# Natural Products-Derived Chemicals: Breaking Barriers to Novel Anti-HSV Drug Development

**DOI:** 10.3390/v12020154

**Published:** 2020-01-29

**Authors:** Jakub Treml, Markéta Gazdová, Karel Šmejkal, Miroslava Šudomová, Peter Kubatka, Sherif T. S. Hassan

**Affiliations:** 1 Department of Molecular Biology and Pharmaceutical Biotechnology, Faculty of Pharmacy, University of Veterinary and Pharmaceutical Sciences Brno, Palackého tř. 1946/1, 612 42 Brno, Czech Republic; jakub.treml@gmail.com; 2 Department of Natural Drugs, Faculty of Pharmacy, University of Veterinary and Pharmaceutical Sciences Brno, Palackého tř. 1946/1, 612 42 Brno, Czech Republic; gazdova.marketa@seznam.cz (M.G.); karel.mejkal@post.cz (K.Š.); 3 Museum of Literature in Moravia, Klášter 1, 664 61 Rajhrad, Czech Republic; sudomova@post.cz; 4 Department of Medical Biology, Jessenius Faculty of Medicine, Comenius University in Bratislava, 036 01 Martin, Slovakia; peter.kubatka@uniba.sk; 5 Division of Oncology, Biomedical Center Martin, Jessenius Faculty of Medicine, Comenius University in Bratislava, 036 01 Martin, Slovakia; 6 Department of Applied Ecology, Faculty of Environmental Sciences, Czech University of Life Sciences Prague, Kamýcká 129, 6-Suchdol, 165 21 Prague, Czech Republic

**Keywords:** herpes simplex virus infection, bioactive natural products, drug resistance, drug development, antiherpetic drugs, preclinical and clinical studies, mechanisms of action

## Abstract

Recently, the problem of viral infection, particularly the infection with herpes simplex virus type 1 (HSV-1) and type 2 (HSV-2), has dramatically increased and caused a significant challenge to public health due to the rising problem of drug resistance. The antiherpetic drug resistance crisis has been attributed to the overuse of these medications, as well as the lack of new drug development by the pharmaceutical industry due to reduced economic inducements and challenging regulatory requirements. Therefore, the development of novel antiviral drugs against HSV infections would be a step forward in improving global combat against these infections. The incorporation of biologically active natural products into anti-HSV drug development at the clinical level has gained limited attention to date. Thus, the search for new drugs from natural products that could enter clinical practice with lessened resistance, less undesirable effects, and various mechanisms of action is greatly needed to break the barriers to novel antiherpetic drug development, which, in turn, will pave the road towards the efficient and safe treatment of HSV infections. In this review, we aim to provide an up-to-date overview of the recent advances in natural antiherpetic agents. Additionally, this paper covers a large scale of phenolic compounds, alkaloids, terpenoids, polysaccharides, peptides, and other miscellaneous compounds derived from various sources of natural origin (plants, marine organisms, microbial sources, lichen species, insects, and mushrooms) with promising activities against HSV infections; these are in vitro and in vivo studies. This work also highlights bioactive natural products that could be used as templates for the further development of anti-HSV drugs at both animal and clinical levels, along with the potential mechanisms by which these compounds induce anti-HSV properties. Future insights into the development of these molecules as safe and effective natural anti-HSV drugs are also debated.

## 1. Introduction

Infection with herpes simplex virus (HSV) has been recognized since antiquity in humans, however, the first in vitro cultivation of HSV was assayed in 1925 [1]. Since 1968, herpes simplex type 1 (HSV-1) and herpes simplex virus type 2 (HSV-2) have been distinguished from each other by different clinical manifestations and tropism [2]. HSV belongs to Herpesviridae, which is a broad family of enveloped-DNA viruses that induce numerous clinically substantial syndromes in both adults and neonates. Several factors including viral entrance, the nature of the disease, and degree of host immune competency could affect the induced syndromes [3,4]. HSV-1 is generally associated with oral or facial infection and encephalitis, while HSV-2 is accountable for genital herpes, which is an important sexually transmitted disease [5]. Moreover, infection with HSV-2 can cause recurrent, painful genital lesions and is often connected with negative psychosocial consequences such as shame, anxiety, and depression. Furthermore, infection with HSV-2 was observed to be a high-risk factor for potential HIV infection, as well as invasive cervical carcinoma [6]. Several reports have declared that HSV is involved in various ocular diseases, including stromal keratitis, endotheliitis and neurotrophic keratopathy [4,5,6,7]. The current existant treatment of HSV infection relies mainly on the use of acyclovir (ACV) and related synthetic nucleoside analogs. Unfortunately, the rigorous utilization of these drugs has led to the establishment of undesirable effects as well as drug-resistant strains [8,9]. Although imperative efforts were taken to develop a vaccination, no vaccines have been validated or marketed for effective prevention of the infection to date. Therefore, the development of new antiviral medications has earned much attention in recent decades [10,11]. While many anti-HSV drugs have already been developed and engaged in the treatment of HSV infections, the search for different sources of anti-HSV drugs is a great task for many researchers and healthcare providers to conquer challenges with drug resistance [12]. Thus, it is an important concern to open new gates to search for new therapeutic agents that perform with different mechanisms of action than nucleoside analogs. Nature is a very rich source of these molecules.

## 2. Epidemiology and Pathogenesis of HSV Infection 

**Figure 1:**
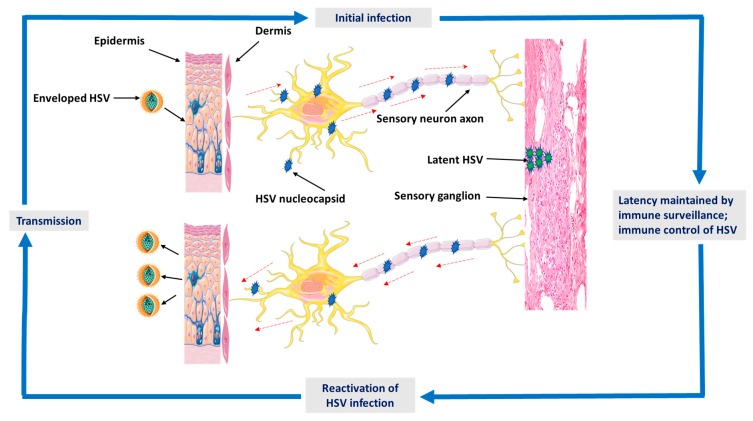
A graphical illustration shows the epidemiology and pathogenesis of herpes simplex virus (HSV) infection. Detailed descriptions are discussed in Section 2.

It’s acknowledged that HSV endures for the lifetime of the host in the form of latent infection in the peripheral neurons [13]. After infection begins, reactivation can be systematically triggered by re-entering the lytic phase of replication to create a progeny virus for spreading [14]. However, during latent infection, the viral lytic genes are largely down-regulated, and their promoters are joined into repressive heterochromatin (Figure 1). Consequently, reactivation necessitates viral lytic gene expression to be created by silenced promoters in the absence of viral proteins [15]. During primary infection, HSV penetrates through breaks in the skin or mucosa and subsequently attaches to and accesses epithelial cells and starts replication. It’s taken up by free sensory nerve endings placed at the dermis, and the nucleocapsid containing the viral genome is transferred by retrograde axonal flow to the nucleus in the sensory ganglion [16,17]. Skin symptoms include vesicular lesions on an erythematous base. Lesions drive to the focal damage of the epithelial layer and a widespread infiltrate of inflammatory cells elaborates in the surrounding rim and the underlying dermal layer [15,18]. It has been estimated that 10–30% of new infections are symptomatic. After recovering from the initial infection, HSV perseveres latently in the sensory ganglion for the life of the host. Regularly, the virus reactivates from the latent state and moves back down the sensory nerves to the skin or mucosal surface [15,19]. Viral shedding can appear either in the presence of lesions as a clinical reactivation or with very moderate or no symptoms as subclinical reactivation. Shedding from mucosal surfaces drives transmission to other sexual partners and, in some cases, infection with HSV can be transferred from mother to infant at delivery [20,21].

## 3. Natural Products-Derived Molecules with Anti-HSV-1 and Anti-HSV-2 Properties

**Figure 2:**
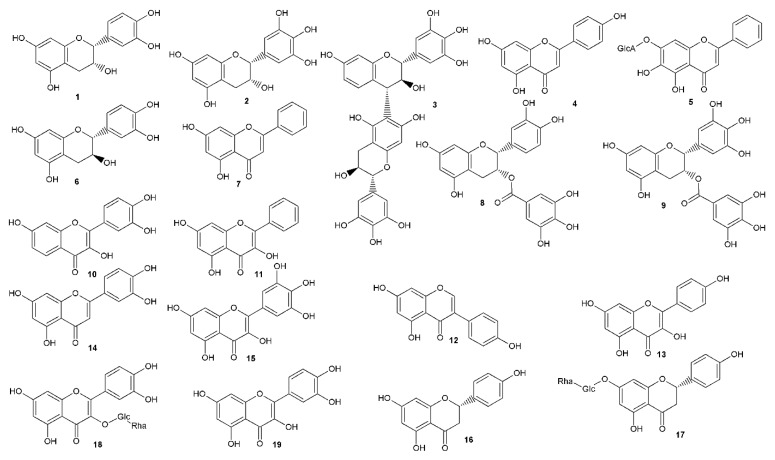

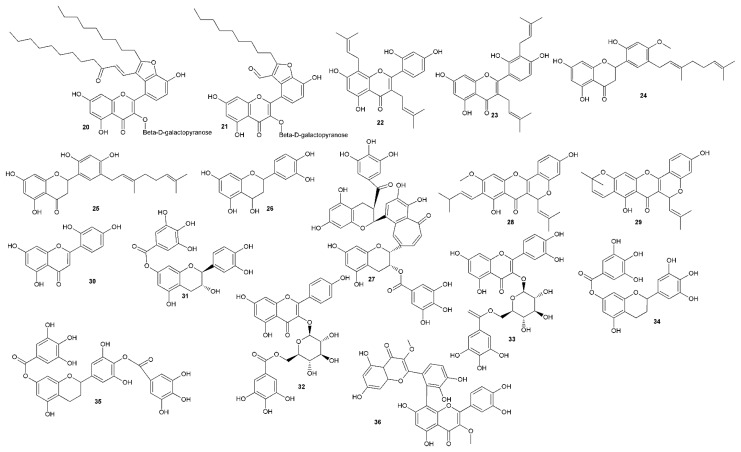

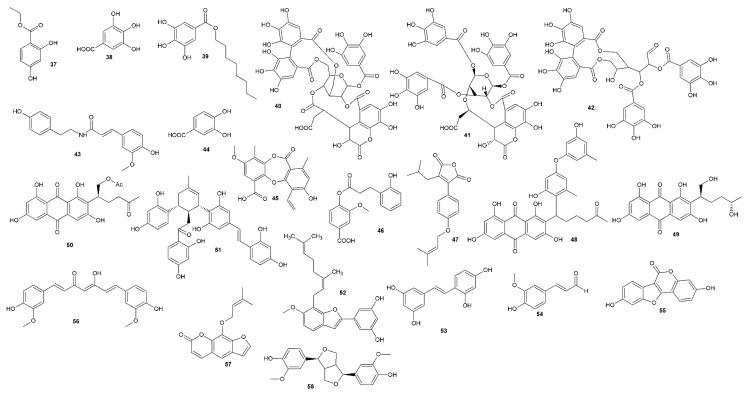
Phenolic compounds with antiherpetic activity.

**Figure 3:**
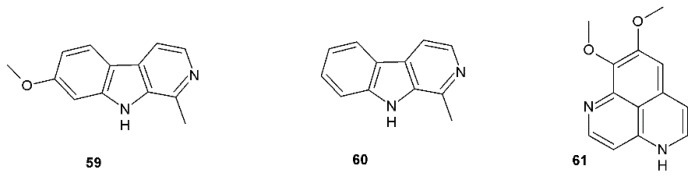
Alkaloids with antiherpetic activity.

**Figure 4:**
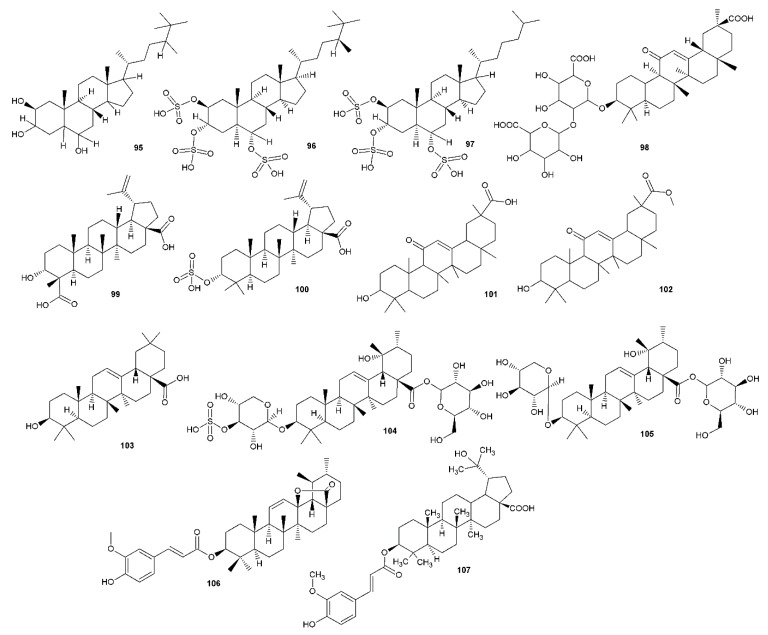
Terpenoid compounds with antiherpetic activity.

**Figure 5:**
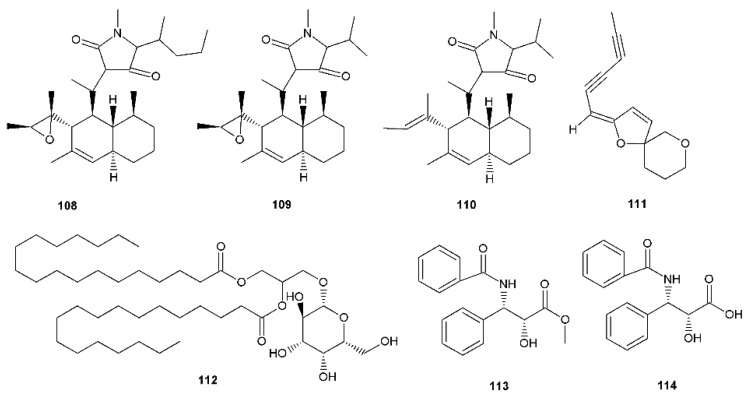
Miscellaneous compounds with antiherpetic activity.

As a part of our ongoing search for natural compounds that are effective against HSV infection, we tried to evaluate progress by reviewing the compounds showing promising anti-herpetic activities. The reviews of the literature covering this area were published previously [22,23,24], with the latest in 2015 [25]. Thus, we followed the latest information and gathered approximately 83 literature sources, which were not included in these papers, showing natural compounds with anti-HSV activity. The SciFinder database was used to cover this area of the published literature and selected data for compounds obtained by the search are presented in Table 1, Table 2, Table 3, Table 4, Table 5 and Table 6 and Figure 2, Figure 3, Figure 4 and Figure 5.

## 4. Natural Products Targeting Enzymes Implicated in HSV Replication

Over the past few decades, structural and mechanistic enzymology played a central role in virology research, where a wide range of enzymes that play a vital role in viral replication, viral transcription or have an impact on the pathogenesis of infection have become imperative drug targets for therapeutic intervention [117,118]. Recently, Čulenová et al. [34] have isolated phenolic compounds from *Morus alba* root bark, kuwanon C (**22**), kuwanon T (**23**), kuwanon U (**24**) and ethyl 2,4-dihydroxybenzoate (**37**) with clear inhibitory action against HSV-1, with IC_50_ values ranging from 0.64 to 1.93 µ/mL, while kuwanon E (**25**) and mulberrofuran B (**52**) inhibited effectively the replication of HSV-2, with EC_50_ values of 0.93 and 1.61 µg/mL, respectively. Molecular docking analysis outcomes proved the effects of the active compounds by targeting the HSV-1 DNA polymerase and HSV-2 protease (proposed as competitive inhibitors), which are crucial enzymes that display an important role in the HSV replication cycle.

**Figure 6:**
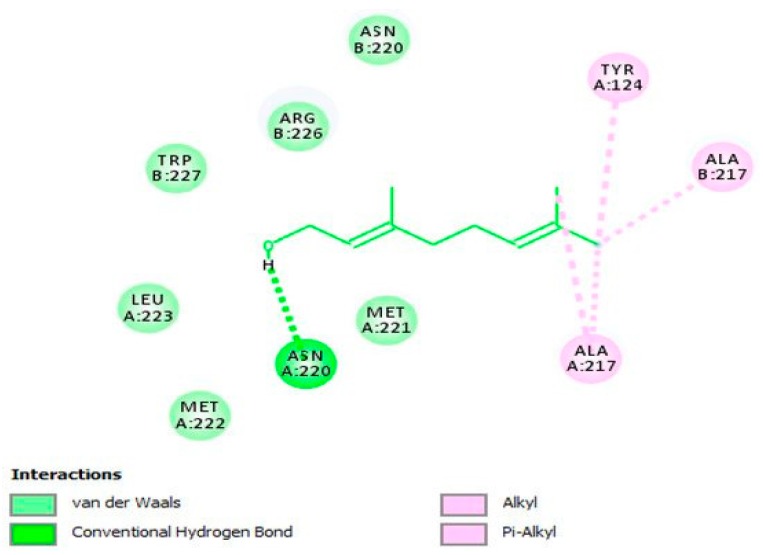
The two-dimensional (2D) interaction diagram of **62** in the active cavity of HSV-2 protease. Only those amino acid residues implicated in the enzyme stabilization are exposed. Hydrogen bonding and several substantial interactions with amino acid residues are displayed. This figure and its description have been adapted from Hassan et al. [57] with permission, as the article has been published by an MDPI publisher and licensed under an open access Creative Commons CC BY 4.0 license.

Geraniol (**62**), a monoterpenoid active compound detected in *Thymus bovei* Benth. essential oil has shown to possess obvious inhibitory effects on HSV-2 replication (EC_50_ = 1.92 µg/mL; SI = 109.38) compared with that of standard ACV (EC_50_ = 1.94 µg/mL; SI = 108.25). This substance, in a molecular docking analysis, has proved to bind to the active site of HSV-2 protease as a competitive inhibitor, and hence uncovered the potential mechanism of action behind the antiherpetic properties against HSV-2 (Figure 6) [57].

**Figure 7:**
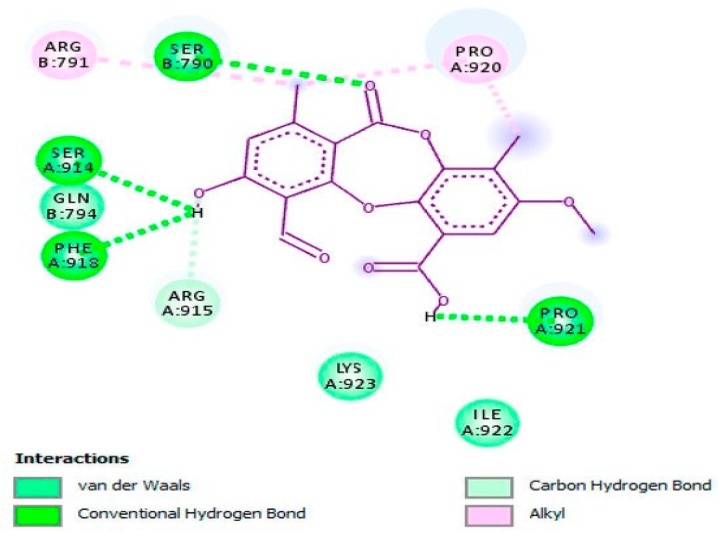
Molecular interaction of psoromic acid (PA, **45**) with the active site of HSV-1 DNA polymerase. Amino acid residues involved in HSV-1 DNA polymerase stabilization along with the hydrogen bonding and other essential interactions for enzyme inactivation are presented. The key functional groups of PA that are responsible for anti-HSV-1 DNA polymerase activity are depicted. This figure and its description have been adapted from Hassan et al. [46] with permission, as the article has been published by an MDPI publisher and licensed under an open access Creative Commons CC BY 4.0 license.

**Figure 8:**
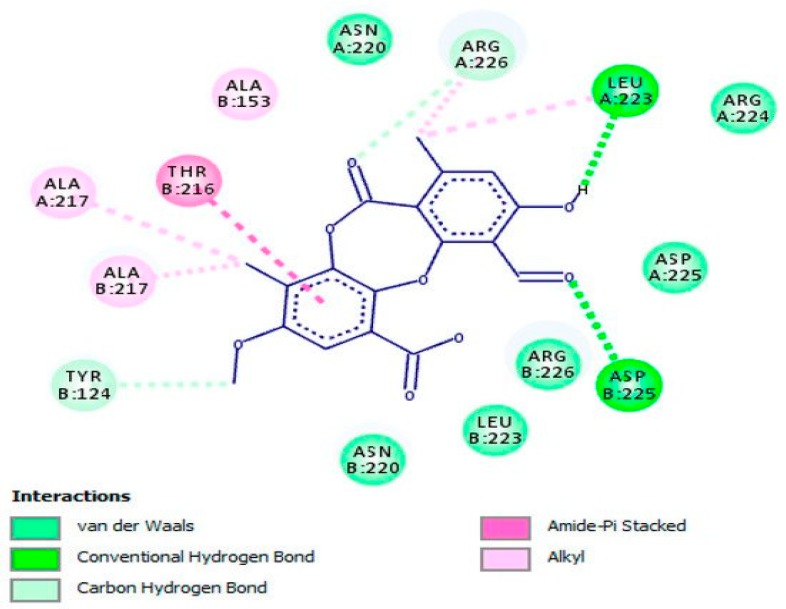
Molecular interaction of psoromic acid (PA, **45**) with the active site of HSV-2 protease. Amino acid residues involved in HSV-2 protease stabilization along with the hydrogen bonding and other essential interactions for enzyme inactivation are illustrated. Significant functional groups of PA that account for the inhibitory action against HSV-2 protease are presented. This figure and its description have been adapted from Hassan et al. [46] with permission, as the article has been published by an MDPI publisher and licensed under an open access Creative Commons CC BY 4.0 license.

In another study, psoromic acid (**45**), a bioactive, lichen-derived molecule, was tested for its inhibitory action against HSV-1 and HSV-2 [46]. The results advocated that this molecule effectively inhibited HSV-1 (IC_50_ = 1.9 μM; SI: 163.2) and HSV-2 (EC_50_ = 2.7 μM; SI: 114.8) replication compared with that of ACV (for HSV-1 IC_50_ = 2.6 μM; SI: 119.2 and for HSV-2 EC_50_ = 2.8 μM; SI: 110.7). Also, the inhibition potency of **45** was enhanced through a combination with ACV as a combinatory treatment. Further, the potential mechanism of action against HSV-1 was revealed by in vitro and in silico assays (Figure 7) via inhibiting the HSV-1 DNA polymerase. In an in vitro assay, **45** was proved to be a non-nucleoside inhibitor as well as a competitive inhibitor of the HSV-1 DNA polymerase with respect to dTTP incorporation (IC_50_ = 0.7 μM; inhibition constant (*K*_i_) = 0.3 μM) compared with reference drugs aphidicolin (IC_50_: 0.8 μM; *K*_i_: 0.4 μM) and ACV triphosphate (ACV-TP) (IC_50_: 0.9 μM; *K*_i_: 0.5 μM). Additionally, molecular docking investigation has revealed the potential mechanism underlying the anti-HSV-2 property of **45** by targeting HSV-2 protease (competitive inhibitor) (Figure 8).

## 5. General Discussion

In general, from the data analysis, we cannot merely conclude with any broad recommendation for further phytochemical research on specific plant family or genus, just some limited hints connected to specific groups of compounds or plant species. First, we have to mention that there is a relative lack of information concerning in vivo testing of compounds assayed in the Vero cell model system against HSV, as described, for example, here [119]. The methodology for testing in vitro anti-HSV activity is commonly based on the assays using the Vero cell line (kidney epithelial cells extracted from an African green monkey (*Chlorocebus* sp.). Vero cells are widely acknowledged to be well-suited for testing antiviral activity, as these cells do not secrete interferon α or β as a response to viral infection, while possessing the INF-α/β receptors, and therefore behave normally after the addition of exogenous interferon [120]. The overall stability and susceptibility of Vero cells to many pathogens, including HSV, makes these cells a very useful tool for testing new potential anti-HSV compounds.

The methodology for testing the anti-HSV activity used in the covered literature search is relatively uniform, allowing the detection of potential hits and finding candidates for antiviral research [121]. The main methods used are analyses of the viability of infected and non-infected cells, the plaque reduction assay, virus cytopathic effect monitoring [122], real-time PCR, quantification of intracellular viral DNA load [123] and the following calculation of selectivity indices. Virus multiplication can also be monitored by ELISA analysis of antigen expression in cell culture. Modifications of these methods, using the sophisticated timing of anti-HSV drug candidate application and further analysis, can give additional information about HSV attachment and penetration to cells [124,125].

The very common therapeutic standard used as the positive control of anti-HSV assays is acyclovir [126]. As it is evident from our literature search and other materials, both HSV-1 and HSV-2, including clinical strains, are sensitive to acyclovir when propagated in Vero cells, with IC_50_ values at low-micromolar concentrations (or micrograms per mL) and selectivity indices reaching values up to 1000 or greater.

According to our literature research, there is an interest in finding new or alternative anti-HSV compounds, represented, for example, by the above-mentioned review published in 2015 [25]. We organized an additional search for anti-HSV natural compounds and gathered information about approximately 100 low-molecular secondary metabolites, obtained from both plants and marine organisms, and also high-molecular polymers represented by a number of sulfated polysaccharides, mainly from marine organisms (algal compounds) and peptides of mainly microbial origin.

Within the compounds mentioned, the most frequent groups with anti-HSV properties are groups of phenolic compounds, comprising a set of simple phenols, flavonoids (mainly dietary flavonoids) and tannins (Table 1). Based on the results of the concurrent analysis and a comparison with previously summarized reports about anti-HSV-activity [22,23,24,25], we can conclude that tannins possess activity comparable to standard acyclovir. Compounds **40** and **41** show activity almost 20× greater and can possibly prevent the attachment of viral particles to the cells and stop the virus’ penetration into the cell [43]. Similarly, some flavonoid aglycones displayed promising results, showing greater effects than acyclovir and greater selectivity. Moreover, according to our recent findings, we can deduce that flavanols are showing greater activity than flavones. This beneficial effect could be possibly subscribed to the 3-OH hydroxy substitution [27]. Furthermore, the treatment of cells with epicatechin gallate (**8**) and galangin (**11**) before HSV adsorption led to some increase in inhibition as determined, indicating that an intracellular activity against the virus may also be involved.

The dual antiviral and antibacterial activity can be beneficial, for example, in the treatment of oral or labial herpetic lesions, which can be relatively easily complicated by secondary bacterial infections. From Table 1, we can deduce that one of the most active phenolic compounds against HSV-1 was kuwanon T (**23**), with IC_50_ 0.64 μg/mL (corresponding to 1.5 μM) and SI 328.1. Kuwanon T (**23**) has also shown promising antibacterial activity against several Gram-positive bacteria, such as methicillin-resistant *Staphylococcus aureus* (MRSA) and *Enterococcus faecalis*. The MIC values of compound **23** ranged from 4–8 μg/mL which exceeded the activity of standard antibiotics ampicillin and ciprofloxacin [34]. Another promising phenolic compound against HSV-1 is galangin (**11**), with IC_50_ 2.5 μM and SI 400. Further, galangin (**11**) has shown bacteriostatic activity against *S. aureus* (ATCC 25293) with MIC value 32 μg/mL [127]. Another phenolic with equal antiviral activity—naringin (**17**)—showed no inhibitory effect on several Gram-positive and Gram-negative, even at a concentration of 250 μM [128]. This dual ability or disability can therefore be a secondary criterion for the potential use of natural anti-herpetic compounds and further research on their activity.

The terpenoids form a relatively wide group of compounds, represented by a number of different skeletons. Each group of terpenoids—monoterpenes, sesquiterpenes, diterpenes and triterpenes (including steroidal compounds)—gave us at least one positive hint in the search. The least abundant are monoterpenes, that are represented only by cypellocarpin C (**63**) isolated from *E. globulus* (**63**, arising from the combination of monocyclic monoterpene with a methylchromone), geraniol (**64**), here obtained from *T. bovei* essential oil [57], showing effects against HSV-2, both IC_50_ and SI, greater than acyclovir [58], and (+)-rhodonoid C (**64**) (a cross-metabolite of monoterpene and polyketide [59]). In comparison to acyclovir, positive results were also obtained for cucurbitacin B (**94**) [73], meroditerpenes from Brazilian seaweed *Stypopodium zonale* **79** and **80** [67], dollabene diterpenes (**85** and **86**) from brown alga *Dictyota pfaffi* [70], which were inhibiting reverse transcriptase of HSV-1, as well as for halistanol derivatives (**95–97**), a steroidal type compound obtained from Brazilian marine sponge *Petromica citrina* [74] which, interestingly, showed a synergistic effect when tested together with acyclovir, but a lower selectivity index when tested alone. Triterpenic tereticornate (**106**) from *E. globulus*, showed an interesting effect against HSV-1 with an SI slightly better than acyclovir [58].

Cucurbitacin B (**94**) is one of the most potent antiviral triterpenoids (IC_50_ = 0.94 μM and SI = 127.7), as shown in Table 3. This compound is also a very effective antibacterial agent—its MIC values against *S. aureus* and MRSA were found to be 0.20 and 0.12 μg/mL, respectively [73]. However, cypellocarpin C (**63**), an effective terpenoid molecule against HSV-2 with IC_50_ = 0.73 μg/mL and SI > 287.7, did not show any antibacterial activity against several Gram-positive and Gram-negative bacteria [58], and, as in the case of phenolics, this can be a selective criterion for further research.

Polysaccharides, heterogeneous natural compounds with promising anti-HSV activity, were reviewed in 2009 [129]. Many of them were isolated from marine seaweeds, especially Chromophyta (brown algae) and Rhodophyta (red algae), and their anti-HSV activities were evaluated and confirmed recently (as visible in Table 5). From the structural point of view, most of them are sulfated polysaccharides with a different degree of sulphation. The degree of sulphation was found to be important for the anti-HSV effect, however, a question remains around the anticoagulant activity of such compounds. Several studies found no correlation between anticoagulant and antiviral activity of sulphated polysaccharides, and such activity would be clinically important only after absorption of the compound into an organism, not during local application. The benefit of anti-HSV polysaccharides can be observed (when measured and calculated) in their high selectivity index. The examples of promising compounds can be partially cyclized μ/v-carrageenan from red seaweed *Gigartina skottsbergii* [101], sulfated galactans from *Schizymenia binderi* [100], and nostoflan, the acidic polysaccharide from terrestrial cyanobacterium *Nostoc flagelliforme* [106].

The last separated reviewed group of compounds are peptides, obtained from various sources, including bacteria, deep-sea fungi, or edible mushrooms. Griffithsin, isolated from red alga *Griffithsia* (family Wrangeliaceae), appears to be very effective against HSV-2, with effects at submicromolar concentrations. Furthermore, griffithsin can be possibly combined with carrageenan and effectively used topically in vivo [115]. Among the potent antiviral peptides against HSV-1 is also melittin, with IC_50_ 1.35 μM and SI 6.3. This peptide acts also as antibacterial—when MRSA was treated with melittin at a concentration of 25 µg/mL, the total number of bacteria decreased by ~2.5–3 log CFU [130].

**Figure 9:**
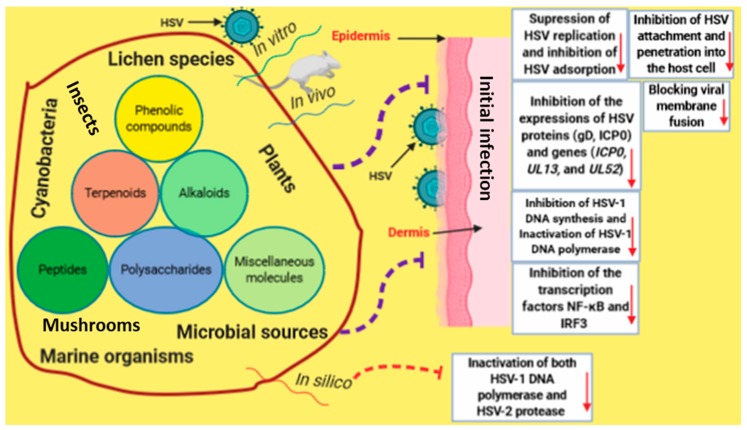
An infographic illustrates the potential mechanisms by which bioactive natural products induce antiviral properties against HSV infection.

From the reviewed articles, all potential mechanisms by which natural products-derived chemicals induced anti-HSV properties have been documented and highlighted, as shown in Figure 9. In the reviewed articles, the majority of assays were basically performed to evaluate the concentration of test compounds necessary to reduce the number of plaques formed in cells and to calculate the selectivity index from the corresponding cytotoxic effect of the test compound on Vero cells. For some compounds, authors performed additional assays to gain deeper insight into the mechanism of action. As an example, chebulagic acid (**40**) and chebulinic acid (**41**) were observed to prevent the attachment and penetration of HSV-2 into Vero cells [43]. Curcumin (**56**) was detected to inhibit HSV adsorption and replication [51], while houttuynoid A (**21**) was noted to block viral membrane fusion [32]. Another good example is the research on prenylated phenol kuwanon X (**51**) [50]. Compound **51** did not inactivate cell-free HSV-1 but inhibited the cellular adsorption and penetration of HSV-1 viral particles. Following viral penetration, **51** reduced the expression of HSV-1 *IE* and *L* genes and decreased the synthesis of HSV-1 DNA. Furthermore, **51** inhibited the HSV-1-induced nuclear factor (NF)-κB activation through blocking the nuclear translocation and DNA binding of NF-κB. The study of Lee et al. [28] gave some insight into the effect of flavonoids, showing the ability of quercetin (**19**), a “prototype” of flavonoid, to inhibit the expressions of HSV proteins (gD, ICP0) and genes (*ICP0*, *UL13*, *UL52*), and specifically suppress the expression of TLR-3 and inhibit the transcription factors NF-κB and IRF3 [28]. The antiviral activity of halistanol derivatives (**96** and **97**) against HSV-1 is enabled by the inhibition of viral particles’ attachment and penetration; the virucidal effect was also observed. Further analysis showed changes in the levels of proteins ICP27 and the gD of HSV-1. These compounds also act synergistically or with acyclovir [74].

## 6. Take-Home Messages

Based on the collected data obtained from the reviewed articles, we may summarize the most promising bioactive natural products that could be used as templates for the further development of anti-HSV drugs through the preparation of analogs using chemical modification processes such as total or semi-synthesis along with combinatorial synthesis, especially with nanoparticles (Table 7). It should be emphasized that we selected bioactive natural products based on the mechanisms of action or types of inhibition induced (against the replication of HSV and its associated steps, or the enzymes involved in the HSV replication cycle). Additionally, these compounds were also selected based on their structure–activity relationship (SAR) that indicates functional groups, which are accountable for the enhanced anti-HSV activity. Based on the above-mentioned selection criteria, where the mechanisms of action, types of inhibition, and SAR are highlighted, we might aid medicinal chemists in the design and synthesis of novel and potent compounds useful for the development of anti-HSV drugs.

## 7. Concluding Remarks and Future Insights

Currently, there are no effective licensed vaccines available for the treatment of herpesviruses infections, and financial support for their development is running short. Studies on novel anti-HSV activities remain a crucial area in drug discovery, since the currently used medications have failed to induce an effective treatment due to the establishment of drug resistance, and there are still a lot of challenges to developing new antiherpetic drug candidates. Therefore, there is an urgent demand to search for new sources that provide less resistance and reduce unwanted effects. Natural products, as a vast source of biologically active molecules, have proven to induce promising inhibitory activities against HSV infection, and hence, in this paper, we highlighted and summarized exclusively the recent investigations on the most promising compounds derived from various natural origins that can be used as promising and effective antivirals for the treatment of diseases caused by HSV; these are in vitro and in vivo studies based on several assay systems. Additionally, the data depicted in this paper demonstrate a notable impact of structural variations, as well as the analysis of proposed structure–activity relationships, and disclosed that the inhibitory activity profile of natural-derived molecules relies upon the position and nature of their substituents. Despite relatively few isolated antiherpetic agents from natural sources advancing to become clinically successful drugs, these unique compounds could be applied as models for the preparation of analogs using chemical modification procedures such as total or combinatorial synthesis, or the alteration of biosynthetic pathways. More research in this field is greatly needed to achieve the design and optimization of potent and selective antiherpetic drugs with promising levels of activity, reduced adverse effects, low toxicity, and enhanced stability. It is known that clinically used antiherpetic drugs do not heal the disease while modifying the clinical course of the infection by suppressing viral replication and subsequent epithelial damage. Thus, there is an imperative need for comprehensive management of HSV infections based on the obstruction of transmission, suppression of recurrence, viral shedding and complications, and modification of clinical, and promotion of treatment, courses. Moreover, the use of natural products with an accepted level of activity against HSV in combination with synthetic nucleoside analogs (as a combinatory treatment) is another valuable option for the therapy of HSV infection; however, these studies are still limited or have yet to be validated. Therefore, all levels of research, including basic-, clinical-, and population-levels, require continued financial support to promote the development and implementation of effective natural anti-HSV drugs with proper pharmacokinetics, pharmacodynamics, hydrolytic stability, and free toxicological profiles (all these assessments should be taken into consideration with all administered forms of the evaluated drug).

## Figures and Tables

**Table 1:** The overview of phenolic compounds with anti-HSV activity.

	Compound	Antiherpetic and Cytotoxicity Assays, Strains, Cells, and Reference Agents	Results	Additional Information	Source
**Flavonoids**	**Epicatechin (1)**	MTT cell viability ACV SI = 4.5	HSV-1, SI = 6.0 (29-R)	Dietary phenolics	[26]
	**Epigallocatechin (2)**		HSV-1, SI = 5.2 (KOS), 12.8 (29-R)		
	**Robinetinidol(4α-6)gallocatechin (3)**		HSV-1 SI = 5.2 (KOS), 5.0 (29-R)		
	**Apigenin (4)**	CPE, PRA, YRA ACV for HSV-1 EC_50_ = 50 μM, SI = 10; HSV-2, EC_50_ = 50 μM, SI = 10	HSV-1, EC_50_ = 5 μM, SI = 50; HSV-2, EC_50_ N/A, SI N/A	Dietary phenolics, green tea, propolis, some flavonoid rich medicinal plants. Flavanols and flavonols appear to be more active than flavones. Furthermore, treatment of Vero cells with ECG (**8**) and galangin (**11**) before virus adsorption led to a slight enhancement of inhibition, indicating that an intracellular effect may be involved.	[27]
	**Baicalin (5)**		HSV-1 EC_50_ = 5 μM, SI= 200; HSV-2 EC_50_ N/A, SI N/A		
	**Catechin hydrate (6)**		HSV-1, EC_50_ = 4 μM, SI = 250; HSV-2, EC_50_ N/A, SI N/A		
	**Chrysin (7)**		HSV-1 EC_50_ = 2.5 μM, SI= 4; HSV-2 EC_50_ N/A, SI N/A		
	**Epicatechin (1)**		HSV-1, EC_50_ = 2.5 μM, SI = 40; HSV-2, EC_50_ = 35 μM, SI = 2.9		
	**Epicatechin gallate (8)**		HSV-1, EC_50_ = 4 μM, SI = 125; HSV-2, EC_50_ = 63 μM, SI = 7.9		
	**Epigallocatechin (2)**		HSV-1, EC_50_ = 2.5 μM, SI = 100; HSV-2, EC_50_ N/A, SI N/A		
	**Epigallocatechin gallate (9)**		HSV-1, EC_50_ = 2.5 μM, SI = 40; HSV-2, EC_50_ N/A, SI N/A		
	**Fisetin (10)**		HSV-1 EC_50_ 2.5 μM, SI = 40; HSV-2 EC_50_ N/A, SI N/A		
	**Galangin (11)**		HSV-1 EC_50_ 2.5 μM, SI = 400; HSV-2 EC_50_ N/A, SI N/A		
	**Genistein (12)**		HSV-1 EC_50_ 5 μM, SI = 50; HSV-2 EC_50_ 50 μM, SI = 5		
	**Kaempferol (13)**		HSV-1 EC_50_ 15 μM, SI = 3.3; HSV-2 EC_50_ N/A, SI N/A		
	**Luteolin (14)**		HSV-1 EC_50_ 5 μM, SI = 20; HSV-2 EC_50_ N/A, SI N/A		
	**Myricetin (15)**		HSV-1 EC_50_ 5 μM, SI = 20; HSV-2 EC_50_ N/A, SI N/A		
	**Naringenin (16)**		HSV-1 EC_50_ 4 μM, SI = 187.5; HSV-2 EC_50_ 22.5 μM, SI 33.3		
	**Naringin (17)**		HSV-1 EC_50_ 2.5 μM, SI = 400; HSV-2 EC_50_ N/A, SI N/A		
	**Rutin (18)**		HSV-1 EC_50_ 5 μM, SI = 2000; HSV-2 EC_50_ N/A, SI N/A		
	**Quercetin (19)**		HSV-1 EC_50_ 5 μM, SI = 20; HSV-2 EC_50_ 35 μM, SI = 2.9		
	**Quercetin (19)**	Raw 264.7 and Vero cells, HSV-1 PRA, Western blot analysis, quantitative RT-PCR	Reduction in plaque formation of 90% at 30 µg/mL	Inhibition of the expressions of HSV proteins (gD, ICP0) and genes (*ICP0*, *UL13*, *UL52*). Specific suppression of the expression of TLR-3, inhibition of transcriptional factors NF-κB and IRF3.	[28]
	**Epigallocatechin gallate (9)**	IP (%)	IP: 100%	Dietary phenolic, green tea component	[29]
		% PFU	At 1 μM cca 40%, at 5 µM cca 5%		[30]
	**Houttuynoid M (20)**	PFA ACV IC_50_ 0.15 μM; SI>1333	IC_50_ 17.72 μM; SI> 11.29 IC_50_ 12.42 μM; SI> 16.10	*Houttuynia cordata*	[31]
	**Houttuynoid A (21)**				
		1. β-galactosidase assay - the activity of enzyme measured in cell lysates 2. PRA 3. Progeny HSV-1 yield assay - effect on HSV-1 multiplication	1. HSV-1 (F) IC_50_ 23.50 ± 1.82 μM, CC_50_ 166.36 ± 9.27 μM 2. HSV-1 (F) IC_50_ of 21.08 μM 3. HSV-1 (F) multiplication reduced by 100% at 75 μM	Possible mechanism—blocking viral membrane fusion	[32]
	**Genistein (12)**	Vero cells, HSV-1 (KOS), HSV-1 (29 R), HSV-2 (333) PRA ACV: IC_50_ 2.44 μM, SI >1818 (KOS), NA (29 R), IC_50_ 3.30 μM, SI >303 (333)	IC_50_ (μM); SI: HSV-1 (KOS)/HSV-1 (29 R)/HSV-2 (333) 14.02, 3.88/7.76, 7.01/14.12, 6.95	Isoflavonoid, soya beans, alfalfa	[33]
	**Kuwanon C (22)**	Vero cells, HSV-1 PRA ACV IC_50_ 1.45 μg/mL; SI 144.8	HSV-1 IC_50_ 0.91 ± 0.43 μg/mL; SI 230.8	In silico analysis along with antibacterial and anti-inflammatory effects	[34]
	**Kuwanon T (23)**		HSV-1 IC_50_ 0.64 ± 0.52 μg/mL; SI 328.1		
	**Kuwanon U (24)**		HSV-1 IC_50_ 1.93 ± 1.13 μg/mL; SI 108.8		
	**Kuwanon E (25)**	Vero cells, HSV-2 TRA ACV IC_50_ 1.65 μg/mL; SI 127.3	HSV-2 IC_50_ 1.61 ± 0.31 μg/mL; SI 130.4		
	**Luteoforol (26)**	Vero cells, HSV-1 (KOS, VR733) CPE as 50% tissue culture infective dose (TCID_50_/50 μL) ACV reduced the titer by 3.16 log_10_ against strain KOS and by 3 log_10_ against strain VR733	Reduced the titer by 2.9 log_10_ against strain KOS and by 3.18 log_10_ against strain VR733	*Hypericum connatum*	[35]
	**Luteolin (14)**	Vero cells, HSV-2 PRA ACV EC_50_ 2.6 μg/mL, SI= 42.53	HSV-2 EC_50_ 22.4 μg/mL, SI = 12.43	Dietary flavonoid	[36]
	**Theaflavin-3,3′-digallate (27)**	Vero cells, HSV-1 PRA Flow cytometry antiviral assay Fluorescence confocal microscopy	EC_50_ 20 μM; SI = 5.625	Green tea	[37]
	**Cycloartocarpin (28)**	Vero cells, HSV-1 (KOS), HSV-2 (186) PRA ACV HSV-1 IC_50_ 1.5 μM; HSV-2 IC_50_ 2.9 μM	HSV-1 IC_50_ 28.2 μM; HSV-2 IC_50_ 23.5 μM	Prenylated phenolics *Morus* spp., *Artocarpus* spp.	[38]
	**Isocyclomorusin (29)**		HSV-1 IC_50_ 30.4 μM; HSV-2 IC_50_ 27.2 μM		
	**Norartocarpetin (30)**		HSV-1 IC_50_ 63 μM; HSV-2 IC_50_ 52.2 μM		
	**Catechin-7-gallate (31)**	Vero cells, HSV-1 CPE ACV CC_50_ >200 ± 0.4 μg/mL	CC_50_ 43.2 ± 2.3 μg/mL	Dietary phenols Low activity, questionable results.	[39]
	**Kaempferol-3-*O*-6′´-*O*-galloyl-*β*-D-glucopyranoside (32)**		CC_50_ 124.1 ± 1.2 μg/mL		
	**Kaempferol (13)**		CC_50_ 76.1 ± 0.2 μg/mL		
	**Quercetin-3-*O*-6´´-*O*-galloyl-*β*-D-glucopyranoside (33)**		CC_50_ 175.6 ± 0.9 μg/mL		
	**Quercetin (19)**		CC_50_ 78.1 ± 0.8 μg/mL		
	**7-*O*-galloyltricetiflavan (34)**	Vero cells, HSV-1 CPE ACV IC_50_ 0.25 μg/mL	IC_50_ 30 μg/mL	*Pithecellobium clypearia* Other viruses tested	[40]
	**7,4′-di-*O*-galloyltricetiflavan (35)**		IC_50_ 20 μg/mL		
	**Strychnobiflavone (36)**	Vero cells, HSV-1 (KOS), HSV-2 (333) PRA Post infection treatment ACV HSV-1 IC_50_ 1.38 μg/mL, SI > 1.449; HSV-2 IC_50_ 3.23 μg/mL, SI > 619	HSV-1 (KOS) IC_50_ 11.82 μg/mL, SI = 22.61; HSV-2 (strain 333) IC_50_ 6.31 μg/mL, SI = 42.33	*Strychnos pseudoquina*	[41]
**Derivatives of phenolic acids**	**Ethyl 2,4-dihydroxybenzoate (37)**	Vero cells, HSV-1 PRA ACV IC_50_ 1.45 μg/mL; SI 144.8	HSV-1 IC_50_ 1.32 ± 0.44 μg/mL; SI 159.1	In silico analysis; antibacterial and anti-inflammatory effects	[34]
	**Gallic acid (38)**	Vero cells, HSV-1 CPE ACV CC_50_ > 200 ± 0.4 μg/mL	CC_50_ 49.8 ± 0.4 μg/mL	Dietary phenols Low activity, questionable results.	[39]
		IP (%)	IP: 100 %	Dietary phenolics	[29]
	**Alkyl derivatives of gallic acid** **Octyl gallate (39)**	HEp-2 and Vero cells, HSV-1 CPE	Octyl gallate directly inactivates HSV-1 (virucidal activity). **39** suppresses both the intracellular multiplication and the release of the virus. **39** selectively accelerates the death of the virus-infected cells. The addition of the compound (**39**), even at 6 h post-infection, completely abolished the formation of progeny virus in the infected cells.	Other viruses tested including HSV-1: Inhibition was enhanced by the compounds with a higher number of carbons in the alkyl moieties, maximum at **12** (lauryl gallate), however, cytotoxicity was increased.	[42]
	**Chebulagic acid (40)**	IPF ACV IC_50_ 29.04 ± 1.04 μg/mL	HSV-2 IC_50_ 1.41 ± 0.51 μg/mL	Dose-dependently potent in vitro direct anti-viral activity. Effective prevention of the attachment as well as penetration of the HSV-2 to Vero cells.	[43]
	**Chebulinic acid (41)**		HSV-2 IC_50_ 0.06 ± 0.002 μg/mL		
	**Tellimagrandin I (42)**	IPF At 0.75 μg/mL ACV completely protected Vero cells against infection	EC_50_ of 2.6 μM for the direct mode, 5.0 μM for the absorption mode.	Ellagitannin—*Cornus* spp., *Eucalyptus* spp., *Melaleuca styphelioides*	[44]
	***N*-*trans*-ferulolyl tyramine (43)**	IP (%)	IP: 100%	Dietary phenolics	[29]
	**Protocatechuic acid (44)**	Vero cells, HSV-2 ACV EC_50_ 1.43 μg/mL, SI= 140	EC_50_ 0.92 µg/mL, SI = 217	Dietary phenolic, metabolite of gut degradation of phenolics	[45]
	**Psoromic acid (45)**	Vero cells, HSV-1, HSV-2 ACV for HSV-1 IC_50_ 2.6 μM; SI 119.2; for HSV-2 EC_50_ 2.8 μM; SI 110.7	HSV-1 IC_50_ 1.9 μM; SI 163.2 HSV-2 EC_50_ 2.7 μM; SI 114.8	Study of synergy with ACV and inhibition of HSV-1 DNA polymerase (in vitro and in silico assays).	[46]
	**Rhinacanthinic acid C (46)**	Vero cells, HSV-2 PRA ACV ED_50_ 14.67 μg/mL	ED_50_ 58.98 μg/mL	*Rhinacanthus nasutus*	[47]
**Anthrones**	**Antrodin A (47)**	Vero cells, HSV-1, HSV-2 PRA ACV HSV-1 IC_50_ 2.1 μg/mL, SI = 61.9, HSV-2 IC_50_ 2.9 μg/mL, SI = 44.8	HSV-1 IC_50_ 5.8 μg/mL, SI= 18.97, HSV-2 IC_50_ 5.5 μg/mL, SI= 20.0	*Antrodia camphorate* Additive effect of **47** with ACV	[48]
	**Aspergilol H (48)**	HSV-1 PRA ACV EC_50_ 3.0 μM	HSV-1 EC_50_ 4.68 μM	Deep-sea fungus *Aspergillus versicolor*	[49]
	**Aspergilol I (49)**		HSV-1 EC_50_ 6.25 μM		
	**Coccoquinone A (50)**		HSV-1 EC_50_ 3.12 μM		
**Stilbenoids and** **2-arylbenzofurans**	**Kuwanon X (51)**	Vero cells, HSV-1 (15577 and clinical strains), HSV-2 (333) PRA ACV IC_50_ 0.1 µg/mL for all strains	HSV-1 IC_50_ 2.2 and 1.5 μg/mL; HSV-2 IC_50_ 2.5 µg/mL	Prenylated phenol, *Morus* spp. **51** did not inactivate cell-free HSV-1 particles but inhibited cellular adsorption and penetration of HSV-1 viral particles. Following viral penetration, **51** reduced the expression of HSV-1 *IE* and *L* genes and decreased the synthesis of HSV-1 DNA. Furthermore, **51** inhibited the HSV-1-induced nuclear factor (NF)-κB activation through blocking the nuclear translocation and DNA binding of NF-κB.	[50]
	**Mulberrofuran B (52)**	Vero cells, HSV-2 TRA ACV IC_50_ 1.65 μg/mL; SI 127.3	HSV-2 IC_50_ 0.93 ± 0.23 μg/mL; SI 225.8	*In silico* analysis; antibacterial and anti-inflammatory effects	[34]
	**Oxyresveratrol (53)**	Vero cells, HSV-1 (KOS), HSV-2 (186) PRA ACV HSV-1 IC_50_ 1.5 μM; HSV-2 IC_50_ 2.9 μM	HSV-1 IC_50_ 42.8 μM; HSV-2 IC_50_ 42.5 μM	Stilbenoid *Morus* spp., *Artocarpus* spp.	[38]
**Other phenolics**	**Coniferyl aldehyde (54)**	Vero cells, HSV-1, HSV-2 ACV HSV-1 EC_50_ 0.8 μg/mL	HSV-1 EC_50_ 6.39 μg/mL, SI = 78.3 HSV-2 EC_50_ 41.2 μg/mL, SI = 12.1	Phenolic, *Quercus suber, Simira glaziovii, S. eleiezeriana*	[51]
	**Coumestrol (55)**	Vero cells, HSV-1 (KOS), HSV-1 (29 R), HSV-2 (333) PRA ACV: IC_50_ 2.44 μM, SI >1818 (KOS), NA (29 R), IC_50_ 3.30 μM, SI >303 (333)	IC_50_ (μM), SI: HSV-1 (KOS)/HSV-1 (29 R)/HSV-2(333) 11.62, 9.6/3.34, 31.52/35.53, 28.14	Coumestan, soya beans, alfalfa	[33]
	**Curcumin (56)**	CPA, PRA, viral adsorption assay, viral penetration assay	At 30 µM, 85% inhibition of HSV-1 and 68% of HSV-2 CPE, PRA 92% for HSV-1 and 88% for HSV-2	*Curcuma longa* Inhibits HSV adsorption and replication	[52]
		Vero cells, HSV-1 CPE ACV CC_50_ > 200 ± 0.4 μg/mL	CC_50_ 49.8 ± 0.4 μg/mL	Dietary phenols Low activity, questionable results	[39]
	**Imperatorin (57)**	Vero cells, HSV-1 CPE ACV – full inhibition of replication of HSV-1 at 250 μg/mL	**57** decreases titer of HSV-1 by 55.6% at 31.25 μg/mL	Furanocoumarin of Apiaceae family	[53]
	**Pinoresinol (58)**	IP (%)	IP: 26%	Dietary phenolics	[29]

HSV-1: herpes simplex virus type 1; HSV-2; herpes simplex virus type 2; ACV: acyclovir; CPE: cytopathic effect; IC_50_: 50% inhibitory concentration; EC_50_: 50% effective concentration; ED_50_: 50% effective dose; CC_50_: 50% cytotoxic concentration; PRA: plague reduction assay; YRA: yield reduction assay; SI: selectivity index = CC_50_/EC_50_ or CC_50_/IC_50_ (cytotox./antiviral); PFU: plaque forming units; IPF: inhibition of plaque formation; TRA: titer reduction assay; MTT assay: 3-(4,5-dimethylthiazol-2-yl)-2,5-diphenyltetrazolium Bromide; Vero cells used for assay if not stated in methods; F, KOS, 29-R—viral strains.

**Table 2:** The overview of alkaloids with anti-HSV activity.

Compound	Antiherpetic and Cytotoxicity Assays, Strains, Cells, and Reference Agents	Results	Additional Information	Source
**Harmine (59)**	Vero cells, HSV-2 PRA ACV CC_50_ and IC_50_ > 3.000 mg/mL and 0.1 μg/mL, respectively, SI > 30.000	CC_50_ and IC_50_ 12.5 μg/mL and 0.3 μg/mL, respectively, SI = 41.6	*Peganum harmala*, *Banisteriopsis caapi*, *Passiflora incarnata*	[54]
	Human foreskin fibroblasts (HFF), HSV-1 (166vVP22-GFP) GFP-based reporter assay Cidofovir at 3 μM reduced to 20%	At 3.3 μM, **59** reduced HSV-1 replication to approx. 50%, at 10 μM to approx. 5%	**59** inhibited viral protein expressed as a dual-specificity tyrosine phosphorylation-regulated kinase inhibitor.	[55]
**Harmane (60)**	Vero cells, HSV-1, HSV-2 PRA ACV HSV-1 EC_50_ 0.8 μg/mL	HSV-1 EC_50_ 4.9 μg/mL, SI = 11.8 HSV-2 EC_50_ 71.8 μg/mL, SI = 24.7	*P. harmala*, *B. caapi*, *P. incarnata*	[51]
**Aaptamine (61) (8,9-dimethoxy-1H-benzo[d,e][1,6]-naphthyridin)**	Vero cells, HSV-1 CPE	EC_50_ 7.0 µg/mL	Marine sponge *Aaptos* spp.	[56]

**Table 3:** The overview of terpenoid compounds with anti-HSV activity.

Compound		Antiherpetic and Cytotoxicity Assays, Strains, Cells, and Reference Agents	Results	Additional Information	Source
**Monoterpenes**	**Geraniol (62)**	Vero cells, HSV-2 ACV (EC_50_ 1.94 µg/mL; SI = 108.25	HSV-2 EC_50_ 1.92 µg/mL SI = 109.38	*Thymus bovei* Benth. essential oil, typical monoterpene of Lamiaceae	[57]
	**Cypellocarpin C (63)**	Vero cells, HSV-1 (KOS), HSV-2 (clinical isolates) PRA, TRI ACV HSV-1 IC_50_ 1.92 ± 0.23 μg/mL, SI >109.4, HSV-2 IC_50_ 1.75 ± 0.33, SI > 120.0	HSV-1 IC_50_ 0.96 ± 0.12, SI > 218.8	**63** is a cross-metabolite of monoterpenic glycoside and a methylchromone), *Eucalyptus globulus*	[58]
	**(+)-rhodonoid C (64)**	Vero cells, HSV-1 CPE ACV IC_50_ 4.2 μM, SI > 100	IC_50_ 80.6 ± 4.7 μM, SI = 2.7	**64** is a cross-metabolite of monoterpene and polyketide *Rhododendron* spp.	[59]
**Sesquiterpenes**	**β-caryophyllene (65)**	Vero cells, HSV-1, HSV-2 (clinical isolates), (HSV-2 ACV-resistant) PRA Time-of-addition assay Virus inactivation assay ACV HS2-2 0.14 μg/mL; SI = 1178; HSV-2 (acyclovir-resistant) EC_50_ 71.84 μg/mL, SI = 2.29	HSV-2 EC_50_ 5.38 μg/mL, SI = 9.10 HSV-2 (acyclovir resistant) EC_50_ 5.02 μg/mL, SI = 9.76	Bicyclic sesquiterpene, common occurrence, for example, cloves	[60]
	**Kellerin (66)**	Vero cells, HSV-1 (KOS) PRA ACV at 2.5 µg/mL, 82% of plaque reduction	**66** at 2.5 µg/mL, 65%	**66** is a cross-metabolite of sesquiterpene and coumarin Gum resin of *Ferula assa foetida* No cytotoxic effect up to 10 µg/mL	[61]
	**Lactarorufin A 8-[*N*-benzoyl-(2′*R*,3′*S*)-3′-phenylisoserinate] (67)**	Vero cells, HSV-1 (MacIntyre strain) CPE ACV IC_50_ 1 µg/mL, SI ˃ 250	HSV-1 IC_50_ 17.3 µg/mL, SI = 16	Taxol-*N*-benzoylphenyl-isoserinates of sesquiterpenoid alcohols and sesquiterpenoids *Lactarius* mushroom	[62]
	**Isolactarorufin 8-[*N*-benzoyl-(2′*R*,3′*S*)-3′-phenylisoserinate] (68)**		HSV-1 IC_50_ 21.9 µg/mL, SI = 17.4		
	**Furandiol 8-[*N*-benzoyl-(2′*R*,3′*S*)-3′-phenylisoserinate] (69)**		HSV-1 IC_50_ 15 µg/mL, SI = 19.3		
	**Isovellerol 13-[*N*-benzoyl-(2′*R*,3′*S*)-3′-phenylisoserinate] (70)**		HSV-1 IC_50_ 7.8 µg/mL, SI = 13.9		
	**5-deoxylactarolid B 8-[*N*-benzoyl-(2′*R*,3′*S*)-3′-phenylisoserinate] (71)**		HSV-1 IC_50_ 3.4 µg/mL, SI = 31.7		
	**Isolactarorufin 8-*epi*-[*N*-benzoyl-(2′*R*,3′*S*)-3′-phenylisoserinate] (72)**		HSV-1 IC_50_ 4.2 µg/mL, SI = 18.4		
	**Alantolactone (73)**	Vero cells, HSV-1 CPE Ribavirin as a positive control	At 10^-6^–10^-8^ g/mL showed an antiviral effect	Sesquiterpene *Inula helenium*	[63]
	**(-)-15-methoxy-3,6-peroxocupar-1-ene (74)**	Vero cells, HSV-1 (KOS strain, VR-1493) PRA ACV at 2.5 μM 96.96%	Anti HSV-1at 10 μg/mL 43.93 %	Sesquiterpene *Schisandra sphenanthera*	[64]
	**(*R*)-6,9-dihydroxy-1-oxo-14-noreudesm-5,7,9-triene (75)**	Vero cells, HSV-2 CPE inhibition method Quantitative PCR	2 log_10_ reduction in HSV-2 yield at conc. 12.5 µM, IC_50_ 6.25 μM	14-Noreudesmane sesquiterpene *Elaeagnus rhamnoides*	[65]
**Diterpenes**	**Simirane A (76)**	Vero cells, HSV-1, HSV-2 PRA ACV HSV-1 EC_50_ 0.8 μg/mL	HSV-1 EC_50_ 4.61 μg/mL, SI = 7.01 HSV-2 EC_50_ 3.73 μg/mL, SI = 8.7	Erythroxylane diterpene, *Simira eliezeriana*	[51]
	**Dodovisnoid D (77)**	Vero cells, HSV-1 CPE ACV IC_50_ 4.2 μM, SI > 100	IC_50_ 5.5 μM, SI = 2.8	Clerodane diterpenes *Dodonaea viscosa*	[66]
	**Dodovisnoid F (78)**		IC_50_ 23.0 μM, SI = 4.7		
	**Atomaric acid (79)**	Vero cells, HSV-1 (ACR-29) CPE ACV EC_50_ 1.2 μM, SI > 716.6	EC_50_ 1.28 µM, SI > 353.1	Meroditerpenes from Brazilian seaweed *Stypopodium zonale* (Dictyotales) Atomaric acid (**79**) and epitanodiol (**80**) may be selectively targeted to HSV-1 replication Low effect on HIV-1 reverse transcriptase	[67]
	**Epitaondiol (80)**		EC_50_ 1.34 µM, SI > 361.9		
	**10-deacetyl-baccatin III (81)**	Vero cells, HSV-1 (MacIntyre strain) CPE ACV IC_50_ 1 µg/mL, SI ˃ 250	HSV-1 IC_50_ 52.7 µg/mL, SI ˃ 9.5	The activity may be associated with their influence on mitotic division. **113** and **114** are included for comparison of effect (non-terpenoid compounds)	[68]
	**Andrographolide (82)**	Vero cells, HSV-1 PRA ACV IC_50_ < 1 µg/mL	IC_50_ 8.28 µg/mL	Ent-labdane diterpenes *Andrographis paniculata* No cytotoxic effect at virucidal concentration.	[69]
	**Neoandrographolide (83)**		IC_50_ 7.97 µg/mL		
	**14-deoxy-11,12-didehydroandrographolide (84)**		IC_50_ 11.1 µg/mL		
	**10,18-diacetoxy-8-hydroxy-2, 6-dolabelladiene (85)**	Vero cells, HSV-1 CPE ACV at 15 µM, 79% of CPE	At 50 µM, 89% of CPE	Dollabene diterpenes brown alga *Dictyota pfaffi* Effect on HIV-1 reverse transcriptase.	[70]
	**10-acetoxy-8,18-di-hydroxy-2,6-dolabelladiene (86)**		At 50 µM, 87% of CPE		
**Steroids**	**Fomitopsin D (87)**	Vero cells, HSV-1 Green fluorescent protein (GFP) expression ACV IC_50_ 2.18 μg/mL	HSV-1 IC_50_ 17 μg/mL	Steroid Fungus *Fomitopsis*	[71]
	**Lyonifoloside A (88)**	Vero cells, HSV-1 (F strain VR 733) CPE ACV EC_50_ 0.41 μM, SI > 244	EC_50_ 11.1 μM, SI = 2.1	9,10-*seco*-cycloartanes **88**–**90**, lanosterol derivatives **91**–**93** *Lyonia ovalifolia*	[72]
	**Lyonifolic acid A (89)**		EC_50_ 3.7 μM, SI = 4.3		
	**Lyofoligenic acid (90)**		EC_50_ 11.1 μM, SI = 5.2		
	**Lyonifolic acid C (91)**		EC_50_ 2.1 μM, SI = 7.6		
	**Lyonifoloside M (92)**		EC_50_ 6.4 μM, SI = 3.0		
	**Lyonifoloside P (93)**		EC_50_ 14.3 μM, SI > 7.0		
	**Cucurbitacin B (94)**	Vero cells, HSV-1 (KOS) PRA Acyclovir IC_50_ 1.74 μM, SI > 132.2	IC_50_ 0.94 μM, SI = 127.7	Cucurbitane steroid Cucurbitaceae	[73]
	**TSH (halistanol (95) rich fraction)**	Vero cells, HSV-1 (KOS) PRA Effects on HSV-1 attachment and penetration ACV IC_50_ 3.45 ± 0.42 μg/mL, SI > 580	IC_50_ 2.87 ± 0.78 μg/mL, SI = 15.53	Brazilian marine sponge *Petromica citrina* (Demospongiae) The observed anti-HSV-1 activity was found to be mediated by the inhibition of virus attachment and by the penetration into Vero cells, the virucidal effect on virus particles, and by the impairment in levels of ICP27 and gD proteins of HSV-1. Synergic effect with acyclovir	[74]
	**Halistanol sulfate (96)**		IC_50_ 5.63 ± 1.37 μg/mL, SI = 2.46		
	**Halistanol sulfate C (97)**		IC_50_ 6.09 ± 1.51 μg/mL, SI = 1.95		
**Pentacyclic triterpenes**	**Glycyrrhizic acid (98)**	HeLa cells, HSV-1 CPE	At 1 and 2 mM, the inhibition ranged from about 78% to 85%	Oleanane triterpene *Glycyrrhizha* spp. 24h pre-treatment strongly enhanced the antiviral activity of **98** (at 2 mM), with a viral inhibition that rise as high as 95%–98%. **98** is a strong inducer of the autophagy activator Beclin 1 (connected to resistance to HSV-1 infection).	[75]
	**Glycyrrhetic acid (101) and its methylester (102)**	Vero cells, HSV-1 strain (KOS) PRA ACV IC_50_ 1.1 ± 0.09 µM, SI> 400	IC_50_ 21.7 ± 0.06 and 8.1 ± 0.2 µM, respectively. SI = 3.9 and > 26, respectively.	Oleanane triterpene *Glycyrrhiza* spp. The hydroxylation at C-21 seems to be responsible for the reduction of anti-HSV-1 activity, the C-29 hydroxy group would eliminate the anti-HSV-1 activity. C-20 methoxy or carboxy groups should be responsible for the enhancement of activity.	[76]
	**3*α*-hydroxylup-20(29)-ene-23,28-dioic acid (99)**	Vero cells, HSV-1 (15577) CPE ACV IC_50_ 0.25 µg/mL, SI > 2000	IC_50_ 31.3 µg/mL, SI = 3.8	Lupane triterpenes *Schefflera heptaphylla* Other viruses tested	[77]
	**3-*epi*-betulinic acid 3-*O*-sulphate (100)**		IC_50_ 20 µg/mL, SI = 5		
	**Oleanolic acid (103)**	Vero cells, HSV-1 (strain F), HSV-2 (strain G) CPE Viral inactivation or virucidal assay Viral penetration assay Time response assay Amplification of viral DNA by PCR ACV HSV-1 EC_50_ 2.1 ± 0.1 μg/mL, SI = 61.9; HSV-2 EC_50_ 2.9 ± 0.1 μg/mL, SI = 44.8	HSV-1 EC_50_ 6.8 ± 1.24 μg/mL, SI = 14.4 HSV-2 EC_50_ 7.8 ± 1.4 μg/mL, SI = 12.6	Common oleanane triterpene Possible inhibition of the early stage of HSV multiplication	[78]
	**Asprellanoside A (104)**	Vero cells, HSV-1 PRA ACV total inhibitory concentration (TIC) 0.0043 mM	TIC 0.14 mM	Sulphur containing triterpenoid saponins *Ilex asprella*	[79]
	**Oblonganoside H (105)**		TIC 0.18 mM		
	**Tereticornate (106)**	Vero cells, HSV-1 (KOS), HSV-2 (clinical isolates) PRA, TRI ACV HSV-1 IC_50_ 1.92 ± 0.23 μg/mL, SI >109.4, HSV-2 IC_50_ 1.75 ± 0.33, SI > 120.0	HSV-1 IC_50_ 0.96 ± 0.12, SI > 218.8	**106** - triterpene *Eucalyptus globulus*	[58]
	***3β-O-trans*-ferulyl-20-hydroxy-lup-28-oic acid (107)**	Vero cells, HSV-1 (F strain VR 733) CPE inhibition method ACV IC_50_ 0.41 ± 0.3 µM, SI > 243.9	IC_50_ 0.71 ± 0.06, SI 5.2	Triterpene *Rhododendron latoucheae*	[80]

**Table 4:** The overview of miscellaneous small molecules with anti-HSV activity.

Compound	Antiherpetic and Cytotoxicity Assays, Strains, Cells, and Reference Agents	Results	Additional Information	Source
**Trichobotrysin A (108)**	Vero cells, HSV-1 PRA ACV IC_50_ 3.50 μM	IC_50_ 3.08 μM	Deep-sea-derived fungus *Trichobotrys effuse* Tetramic acid derivatives	[81]
**Trichobotrysin B (109)**		IC_50_ 9.37 μM		
**Trichobotrysin D (110)**		IC_50_ 3.12 μM		
**(*E*)-2-(2,4-hexa-diynyliden)-1,6-dioxaspiro[4.5]** **dec-3-ene (111)**	Vero cells, HSV-1 (clinical isolate with >99% homology to isolate SK087 US4–6 genes), HSV-2 (clinical isolate >99% homology to isolate 99-62039 US4 gene) CPE, YRA Time-of-addition, adsorption inhibition, virucidal, penetration inhibition assays Macromolecular synthesis inhibition analysis ACV HSV-1 EC50 0.9 μg/mL; SI > 1000 / HSV-2 EC50 0.7 μg/mL; SI > 1000	EC_50_, SI: HSV-1/HSV-2 0.146 μg/mL; > 205 / 0.127 μg/mL; > 236	*Tanacetum vulgare* Spiroketal-enol ether derivative. Mechanism of antiviral activity elucidated on petroleum ether extract and **111** (inhibition of viral gene expression).	[82]
***Monogalactosyl diglyceride (112) and digalactosyl diglyceride (DGDG)***	Vero cells, HSV-1, HSV-2 PRA ACV HSV-1 IC_50_ 0.64 μg/mL and HSV-2 IC_50_ 0.80 μg/mL	HSV-1 IC_50_ 36.00 μg/mL for **112** and 40.00 μg/mL for DGDG, respectively. HSV-2 IC_50_ 41.00 μg/mL for **112** and 43.20 μg/mL for DGDG, respectively.	*Clinacanthus nutans*	[83]
**Methyl (*N*-benzoyl-(2′*R*,3′*S*)-3′-phenylisoserinate) (113)**	Vero cells, HSV-1 (MacIntyre strain) CPE ACV IC_50_ 1 µg/mL, SI ˃ 250	HSV-1 IC_50_ 10.7 µg/mL, SI ˃ 46.7	Taxol derivatives. The activity may be associated with their influence on mitotic division.	[68]
***N*-benzoyl-(2′*R*,3′S)-3′-phenylisoserine (114)**		HSV-1 IC_50_ 21.7 µg/mL, SI ˃ 23		

**Table 5:** The overview of polysaccharides with anti-HSV activity.

Compound	Antiherpetic and Cytotoxicity Assays, Strains, Cells, and Reference Agents	Results	Additional Information	Source
**PSP-B2 polysaccharide from *Prunellae Spica* (*Prunella vulgaris* L.)**	Vero cells, HSV-1, HSV-2 PRA ACV HSV-1 IC_50_ 0.78 µM, HSV-2 1.32 μM	HSV-1 IC_50_ 69 μg/mL HSV-2 IC_50_ 49 μg/mL	No cytotoxicity even at 1600 μg/mL	[84]
***Eucheuma gelatinae* (seaweed) polysaccharide**	Vero cells, HSV-1 PRA ACV EC_50_ HSV-1(strain F), HSV-2 (strain 333), HSV-1 (strain 106), HSV-1 (strain 153), and HSV-1 (strain blue) 0.78, 0.71, 9.60, 21.11, and 23.50 μg/mL, respectively	HSV-1/F, HSV-2/333, HSV-1/106, HSV-1/153, and HSV-1/blue EC_50_ 0.65, 2.12, 1.11, 1.24, and 1.48 μg/mL, respectively	Effect via activity on early HSV-1 infection. Inhibition of viral DNA synthesis.	[85]
**Sulfated polysaccharide SP-III from *Sargassum latifolium***	Vero cells, HSV-1 PRA	SP-III 33% and 81% inhibition at 20 μg/mL and 40 μg/mL, respectively.	Glucuronic acid, mannose, glucose, xylose and fucose.	[86]
**Sulfated polysaccharide SP-2a from *Sargassum patens* (Kütz.) Agardh**	Vero cells, HSV-1 (15577 strain, clinical strain, DM2.1 strain-ACV resistant) PRA Determination of extracellular virucidal activity Time of addition experiment Virus adsorption assay	Inhibition of replication of both the acyclovir-sensitive and -resistant strains of HSV-1, in a dose-dependent manner, EC_50_ 1.5–5.3 μg/mL	Fucose, xylose, mannose, glucose, galactose, galactosamine Extracellular virucidal activity only against the ACV-sensitive strains. This compound might inhibit the attachment of the virus to its host cell.	[87]
**ST-F polysaccharide from marine brown algae *Sargassum trichophyllum***	Vero cells, HSV-2 (UW264 strain) PRA Time of addition experiment	Added to the medium during infection and throughout the incubation (Experiment A) or immediately after viral infection (Experiment B), IC_50_ 18 and 410 μg/mL, respectively. SI > 280 and >12 for A and B, respectively.	(Fucose and galactose) The main antiviral target of ST-F might be virus adsorption and/or penetration step(s) on the host cell surface. Low cytotoxicity	[88]
**SPs -** **sulfated polysaccharides from brown sea algae *Sargassum fluitans* and red sea algae *Solieria filiformis***	Vero cells, HSV-1 Neutral red dye method ACV EC_50_ 15.4 ± 5.6 μg/mL	*S. fluitans* EC_50_ 42.8 ± 4.3 μg/mL and *S. filiformis* EC_50_ 136.0 ± 12 μg/mL Without cytotoxicity (1–200 μg/mL)	The activity observed suggests that the degree of sulfation, molecular weight, and carbohydrate nature of these polysaccharides may affect the activity	[89]
**Polysaccharide fractions C1_p_ and C4_p_ from chlorophyta *Ulva armoricana***	Vero cells, HSV-1 (wild-type strain 17, sensitive to ACV) CPE ACV EC_50_ 0.3 µg/mL	EC_50_ 373.0 ± 20.7 and 320.9 ± 6 µg/mL	Activities correlated to amounts of rhamnose, uronic acids and degree of sulfation.	[90]
**Sp-Am polysaccharide from *Acanthophora muscoides***	Vero cells, HSV-1, HSV-2 CPE	HSV-1 IC_50_ 1.63 μg/mL, SI = 3.5 HSV-2 IC_50_ 3.5 μg/mL, SI = 99.9	Sulfated polysaccharides from marine seaweeds The possible mechanism of the effect - the inhibition of virus adsorption.	[91]
**SP-Gb polysaccharide from *Gracila riabirdiae***		HSV-1 IC_50_ 0.75 μg/mL, SI = 1.25 HSV-2 IC_50_ 82.2 μg/mL, SI = 94.40		
**SP-Sf polysaccharide from *Solieria filiformis***		HSV-1 IC_50_ 0.6 μg/mL, SI = 1.6 HSV-2 IC_50_ 74.9 μg/mL, SI = 97.5		
**PSC polysaccharide from marine seaweed *Sphaerococcus coronopifolius***	Vero cells, HSV-1 (wild type strain 17, sensitive to ACV) CPE Time of addition assay Virus adsorption assay ACV SI ˃ 500	EC_50_ 4.1 μg/mL, SI = 61	Galactose, 3,6-anhydrogalactose, uronic acids, sulfated The adsorption step of HSV-1 to the host cell possible mechanism of action.	[92]
**PBT polysaccharide from marine seaweed *Boergeseniella thuyoides***		EC_50_ 17.2 μg/mL, SI = 14.5		
**Sulfated xylogalactofucans and alginic acids from brown algae *Laminaria angustata***	RC-37 cells, HSV-1 (KOS) PRA Time of addition assay Virus adsorption assay Virucidal assay	IC_50_ 0.21–25 μg/mL, SI ˃ 40 ˃ 3225	Possible inhibiting HSV attachment to cells by direct interaction with viral particles.	[93]
**SU1F1 polysaccharide from green algae *Enteromorpha compressa***	HEp-2 cells, HSV-1 (clinical isolate) PRA Time-of-addition assay Inhibition of adsorption assay Inhibition of penetration assay Virucidal assay Acyclovir IC_50_ 2100 μg/mL, SI = 1.21	IC_50_ 28.25 μg/mL, SI = 35.3	Chemically altered- sulfated ulvan Broad mechanism of action.	[94]
**Sulfated fucoidans (S1-S3) from marine brown alga *Padina tetrastomatica***	Vero cells, HSV-1 (strains F and B2006), HSV-2 (strain MS) PRA Virucidal assay Effect of treatment period on the antiviral activity	HSV-1 and HSV-2 with IC_50_ in range of 0.30–1.05 μg/mL B2006 S3 IC_50_ 0.6 μg/mL	Active during the virus adsorption period Degree of sulfation affects the activity	[95]
**Polysaccharides from brown seaweed *Stoechospermum marginatum***	Vero cells, HSV-1 (strains F, TK^-^ B2006 and filed strains, syncytial variants arising after selection with a natural carrageenan, syn 13-8 and 14-1), HSV-2 (MS) PRA	HSV-1 (F) EC_50_ 1.15-50 μg/mL, SI = ˃20 ˃ 869 HSV-2 (MS) EC_50_ 0.78 μg/mL, SI= 0.57 ˃50 **F3** B2006, Field, 13-8 and 14-1 strains EC_50_ 0.95, 1.52, 4.52 μg/mL, SI ˃1053, ˃658, ˃221, ˃176	Sulfated fucans Active during the virus adsorption period. No direct virucidal activity. No correlation between the antiviral and anticoagulant activity.	[96]
**CiWE CiF3 polysaccharides from brown seaweed *Cystoseira indica***	Vero cells, HSV-1 (strain F), HSV-2 (strain MS) PRA	IC_50_ values in the range of 0.5–2.8 μg/mL	Sulfated fucans Degree of sulfation affects the activity. No correlation between the antiviral and anticoagulant activity.	[97]
**Sulfated polysaccharide (fucoidan) from brown algae *Undaria pinnatifida* (Mekabu)**	Vero cells, HSV-1 (strain HF), HSV-2 (strain UW-268) PRA A) 1 h after the viral infection, B) immediately after infection	HSV-1 IC_50_ and SI A) 2.5 μg/mL, ˃800; B) 14 μg/mL, ˃140 HSV-2 IC_50_ and SI A) 2.6 μg/mL, ˃770; B) 5.1 μg/mL, ˃390	Fucose, galactose Other viruses tested.	[98]
**Polysaccharides (GiWE and F3) from red seaweed *Grateloupia indica***	Vero cells, HSV-1 (strain F, TK^-^ B2006 and filed strains, syncytial variants arising after selection with natural carrageenan, syn 13-8 and 14-1), HSV-2 (MS) PRA	HSV-1 (F) IC_50_ 0.27 μg/mL and HSV-2 (MS), IC_50_ 0.31 μg/mL **F3** B2006, Field, 13-8 and 14-1 strains EC_50_ 0.89, 0.87, 1.06, 0.81 μg/mL, SI ˃ 1123, ˃1149, ˃943, ˃1234 No direct virucidal activity at 40 μg/mL	Sulfated galactans Degree of sulfation affects the activity. Possible ability to interfere with the replication cycle.	[99]
**Polysaccharide from *Schizymenia binderi***	Vero cells, HSV-1 (strains F, TK^−^ (B2006), (Field)), HSV-2 (G) PRA	EC_50_ 0.21-0.76 μg/mL SI > 1000 for all assays No cytotoxicity at 1000 μg/mL	Sulfated galactan Interference with the initial adsorption of viruses to cells, no virucidal activity at 100 μg/mL.	[100]
**Polysaccharide from red seaweed *Gigartina skottsbergii***	Vero cells, HSV-1 (strains F, TK^−^ (B2006), (Field), clinical isolates 1213 LCR/94, 374 LCR/94 and 1180 BE/94), HSV-2 (G, clinical isolate 244 BE/94) PRA	1C_3_ HSV-1 (F) and HSV-2 (G) EC_50_ 0.7 and 0.5 µg/mL, respectively, SI ˃ 1408 and ˃2128 1T_1_ HSV-1 (F) and HSV-2 (G) EC_50_ 0.6 and 0.4 µg/mL, respectively, SI ˃ 1538 and ˃2439 Clinical isolates EC_50_ 0.19–2.18 µg/mL	Carrageenans Lack of anticoagulant activity No virucidal activity, effect on virus adsorption	[101]
**Proteoglycan GLPG from *Ganoderma lucidum* (Agaromycetes)—lingzhi mushroom**	Vero cells, HSV-1, HSV-2 CPE Virus yield inhibition assay	HSV-1 and HSV-2 EC_50_ 48 and 56 µg/mL, respectively, SI ˃ 42 and >36 No cytotoxicity at 2000 µg/mL	Proteoglycan GLPG (carbohydrate: protein ratio of 10.4: 1) The antiviral activity may be due to its inhibiting HSV attachment to cells, in addition, its inhibition of viral penetration would augment its antiviral activity.	[102]
**Polysaccharide RP from *Portulaca oleracea***	Vero cells, HSV-2 (UW268 strain) PRA Time-of-addition assay Virus adsorption and penetration assay	A: RP added during infection and throughout the incubation thereafter B: RP added immediately after viral infection. A: EC_50_ 210 µg/mL, SI = 33 B: EC_50_ 320 µg/mL, SI = 22	Pectic polysaccharide (**RP**) Activity against influenza virus tested on MDCK cells.	[103]
**Polysaccharide SPLCf from *Caesalpinia ferrera***	HEp-2 cells, HSV-1 (clinical isolate) PRA Time-of-addition assay Inhibition of adsorption assay Inhibition of penetration Virucidal activity HSV-1 cell to cell spread assay ACV IC_50_ 2100 μg/mL, SI >1.21	IC_50_ 405 μg/mL, SI > 7.4.	Sulfated polysaccharide SPLCf showed the effect on several stages of the HSV replication—virus adsorption, the effect on virus particles and the expression of viral protein.	[104]
**Polysaccharides (ANP, AAP) from *Acanthopanax sciadophylloides***	Vero cells, HSV-2 (UW264 strain) PRA In vivo anti-HSV-2 effects on female BALB/c mice ACV In vivo: Polysaccharides (1 mg/20 μL) or ACV (0.2 mg/20 μL) administered intravaginally twice per day from 3 days before infection to 7 days post-infection.	**ANP** and **AAP** IC_50_ 52 and 620 μg/mL when added to the medium during infection and throughout the incubation thereafter HSV-2 IC_50_ 67 and 580 μg/mL when added to the medium immediately after infection.	*Acanthopanax sciadophylloides* Virus titers in the vaginal region were decreased by the administration of AAP.	[105]
**Acidic polysaccharide (nostoflan) from terrestrial cyanobacterium *Nostoc flagelliforme***	Vero cells, HSV-1 (HF strain) and HSV-2 (UW268 strain) PRA	A: added during infection and throughout the incubation thereafter B: added immediately after viral infection A: HSV-1 IC_50_ 0.37 μg/mL, SI = 13000 A: HSV-2 IC_50_ 2.9 μg/mL, SI = 2700 B: HSV-1 IC_50_ ˃100 μg/mL, SI = <49 B: HSV-2 IC_50_ 7.7 μg/mL, SI = 1000	Other antiviral activity tested. No anti-thrombin activity.	[106]
**Polysaccharide sulfate fraction from *Caulerpa racemosa***	Vero cells, HSV-1 strain F, TK^-^ B2006 and field strains), HSV-2 G strain) PRA	HSV-1 (strain F, TK^-^ B2006 and field strains) EC_50_ 4.2, 2.4, 2.2 µg/mL, SI ˃ 238, ˃417, ˃454 HSV-2 (strain G) EC_50_ 3.0 µg/mL, SI ˃ 333 No cytotoxic effects up to 1000 µg/mL	Galactose, glucose, arabinose, and xylose as the major components.	[107]

**Table 6:** The overview of peptides with anti-HSV activity.

Compound	Antiherpetic and Cytotoxicity Assays, Strains, Cells, and Reference Agents	Results	Additional Information	Source
**Bacteriocins** (semi-purified)	Vero cells, HSV-1 (strain EK) CPE Viral adsorption assay	Before adsorption IC_50_ 235.6 μg/mL, SI 4.6 (for GEn14) After adsorption IC_50_ 24.0 μg/mL, SI 17.8 (for GEn17)	Bacteria from goat milk *Enterococcus durans* (GEn09, GEn12, GEn14, and GEn17).	[108]
**Subtilosin**	Vero cells, HSV-2 (strain G) PRA Virucidal assay Time-of-addition assay Indirect immunofluorescence assay	At 200 μg/mL a reduction over 99.9% in virus titer EC_50_ 18.2 μg/mL, SI 17.4	*Bacillus amyloliquefaciens* Cyclic peptide Antiviral and virucidal effect. This compound affects late stages of the viral replicative cycle such as viral glycoprotein intracellular transport.	[109]
**Simplicilliumtide J**	Vero cells, HSV-1 PRA ACV IC_50_ 3.0 µM	IC_50_ 14.0 µM	Deep-Sea-Derived Fungus *Simplicillium obclavatum* Cyclic peptides	[110]
**Verlamelin A**		IC_50_ 16.7 µM		
**Verlamelin B**		IC_50_ 15.6 µM		
**Aspergillipeptide D**	Vero cells, HSV-1 (strain 15577, ACV resistant clinical isolates HSV-1-106 and HSV-1-153) CPE ACV IC_50_ 3 µM	HSV-1 (strain 15577) IC_50_ 9.5 µM	Marine gorgonian-derived fungus *Aspergillus* sp. No cytotoxicity at concentrations tested. Aspergillipeptide D showed activity against acyclovir-resistant HSV-1-106 and HSV-1-153.	[111]
**Aspergillipeptide E**		HSV-1 (strain 15577) IC_50_ 19.8 µM		
**RC28** (28.25 kDa) protein	BGMK cells, HSV-1 (KOS) CPE	IC_50_ 0.078 mg/mL, SI > 32	Edible mushroom *Rozites caperata* (*Cortinarious caperata*)	[112]
**Pa-MAP**	Vero cells, HSV-1 Virus titer reduction method Virucidal assay ACV at 20 μg/mL PI 99%	EC_50_ 83 μg/mL, SI ˃ 5	Polar fish *Pleuronectes americanus*	[113]
**Bovine lactoperoxidase**	Vero cells, HSV-1 PRA	At 0.5 mg/mL 100% antiviral effect	Milk hemoprotein	[114]
**Griffithsin**	Vero cells, HeLa cells, HSV-2 (strain G) PrestoBlue cell viability reagent - reading of fluorescence Flow cytometry Inhibition of adsorption assay In vivo HSV-2 murine model	EC_50_ 5.8 μg/mL (230 nM) Griffithsin/carrageenan combination EC_50_ 3.4 ng/mL	Red alga *Griffithsia* A lectin with high affinity for mannose-rich N-linked glycans. Griffithsin may block viral entry by binding to HSV-2 glycoprotein D. The griffithsin/carrageenan combination product but not GRFT or CG alone, reduced HSV-2 vaginal infection in mice when given an hour before challenge.	[115]
**Melittin**	Vero cells, HSV-1 Virus yield inhibition assay	EC_50_ 1.35 μM; SI 6.3	Cationic 26 amino acids peptide isolated from insects—the main component of bee venom	[116]

**Table 7:** Bioactive natural products reported as inducing potent anti-HSV properties.

Chemical Class	Compound	Mechanisms of Action or Types of Inhibition	Structure–Activity Relationship (SAR)
Flavan-3-ol (flavonoid)	**Epicatechin gallate (ECG) (8)**	Inhibition of viral adsorption.	—
Flavonol (flavonoid)	**Galangin (11)**	Inhibition of viral adsorption.	—
Flavonol (flavonoid)	**Quercetin (19)**	Inhibition of the expressions of HSV proteins (gD, ICP0) and genes (*ICP0, UL13, UL52*). Additionally, this molecule suppressed the expression of TLR-3 and inhibited the transcriptional factors NF-κB and IRF3.	—
Flavonoid	**Houttuynoid A (21)**	Blocking viral membrane fusion.	—
Phenolics	**kuwanon C (22), kuwanon T (23), kuwanon U (24), kuwanon E (25), and ethyl 2,4-dihydroxybenzoate (37)**	Inhibition of HSV-1 and HSV-2 replication (in vitro) and inactivation of HSV-1 DNA polymerase and HSV-2 protease (proposed as competitive inhibitors via in silico assay).	Hydroxyl, carbonyl, and methyl groups along with phenyl ring (proposed as functional groups via in silico assays).
Alkyl derivatives of gallic acid	**Octyl gallate (39)**	Inhibition of multiplication of HSV-1 and suppression of formation of virus progeny at early stages (within 6 h post-infection) in the infected cells.	Alkyl moieties.
Tannins	**Chebulagic acid (40) and chebulinic acid (41)**	Avoiding the attachment and penetration of HSV-2 into Vero cells.	—
β-orcinol depsidone, a type of phenolic compound	**Psoromic acid (45)**	Inhibition of HSV-1 and HSV-2 replication and inactivation of HSV-1 DNA polymerase (competitive inhibitor via in vitro and in silico experiments). Also, via in silico assay, inactivates HSV-2 protease (competitive inhibitor).	Hydroxyl, carbonyl, and methyl groups along with phenyl ring (proposed as functional groups via in silico assays).
Stilbene derivative	**Kuwanon X (51)**	Anti-HSV activity through multiple modes of action (impeded cellular adsorption and penetration of HSV-1 viral particles). After viral penetration, this agent decreased the expression of HSV-1 IE and L genes and diminished the synthesis of HSV-1 DNA. Moreover, this molecule prevented the HSV-1-induced nuclear factor (NF)-κB activation via obstructing the nuclear translocation and DNA binding of NF-κB.	—
Flavonoid	**Curcumin (56)**	Inhibition of adsorption and replication of HSV.	Hydroxyl groups (assessed as functional groups).
Alkaloid	**Harmine (59)**	Inhibition of viral protein expression.	—
Monoterpenoid	**Geraniol (62)**	Inhibition of HSV-2 replication (in vitro assay) and inactivation of HSV-2 protease (in silico assay).	Hydroxyl and methyl groups (proposed as functional groups via in silico assay).
Steroids	**Halistanol sulfate (96) and halistanol sulfate C (97)**	Suppression of HSV-1 attachment and penetration into the host cells. These substances also impair the levels of ICP27 and gD proteins of HSV-1.	Sulfate groups (assessed as functional groups).
Triterpene glycoside	**Glycyrrhizic acid (98)**	The compound was detected to be an effective inducer of the autophagy activator Beclin 1, which creates a resistance to HSV-1 replication.	Carboxyl and hydroxyl groups along with sugar moiety (assessed as functional groups).
Triterpenoid	**Methylester of glycyrrhetic acid (102)**	Inhibition of HSV-1 replication.	Methoxy and carboxy groups at C-20 were noted to be responsible for the enhanced inhibitory activity against HSV-1 replication.
Pentacyclic triterpenoid	**Oleanolic acid (103)**	Inhibition of HSV-1 and HSV-2 multiplication at the early stage.	—
Spiroketal-enol ether derivative	**(E)-2-(2,4-hexa-diynyliden)-1,6-dioxaspiro[4.5]** **dec-3-ene (111)**	Suppression of viral gene expression and reduction of viral protein accumulation within infected cells.	—
Taxol derivatives	**Methyl (*N*-benzoyl-(2′R,3′S)-3′-phenylisoserinate) (113) and *N*-benzoyl-(2′R,3′S)-3′-phenylisoserine (114)**	Inhibition of HSV-1 replication (the inhibitory activity might be related to the impact on the mitotic division).	—
Polysaccharides	**Polysaccharides and sulfated polysaccharides**	Multiple mechanisms of action (inhibition of HSV replication, inhibition of virus adsorption, suppression of gene expression, suppression of HSV attachment and penetration into the host cell).	Sugar moieties and sulfate groups.
Cyclic peptide	**Subtilosin**	This antiherpetic agent alters the late stages of the viral replicative cycle such as viral glycoprotein intracellular transport.	—
Peptide	**Griffithsin**	Blocking viral entry by attaching with HSV-2 glycoprotein D.	—

This table digests the most promising bioactive natural products that have been shown to possess potent anti-HSV activity based on their mechanisms of action, types of inhibition, and SAR, which have been displayed in this review. SAR: Structure–activity relationship that signifies functional groups which are responsible for the improved anti-HSV activity. (—): Data not provided in the articles that have been cited in this review.
